# Aptamer Microarrays—Current Status and Future Prospects

**DOI:** 10.3390/microarrays4020115

**Published:** 2015-03-24

**Authors:** Martin Witt, Johanna-Gabriela Walter, Frank Stahl

**Affiliations:** Institut für Technische Chemie, Leibniz Universität Hannover, Callinstrasse 3, 30167 Hannover, Germany; E-Mails: witt@iftc.uni-hannover.de (M.W.); walter@iftc.uni-hannover.de (J.-G.W.)

**Keywords:** aptamer, microarray, multiplex, protein detection, small molecules

## Abstract

Microarray technologies are state of the art in biological research, which requires fast genome, proteome and transcriptome analysis technologies. Often antibodies are applied in protein microarrays as proteomic tools. Since the generation of antibodies against toxic targets or small molecules including organic compounds remains challenging the use of antibodies may be limited in this context. In contrast to this, aptamer microarrays provide alternative techniques to circumvent these limitations. In this article we review the latest developments in aptamer microarray technology. We discuss similarities and differences between DNA and aptamer microarrays and shed light on the post synthesis immobilization of aptamers including corresponding effects on the microarray performance. Finally, we highlight current limitations and future prospects of aptamer microarray technology.

## 1. Introduction

Aptamers are single-stranded oligonucleotides. Depending on their sequence, the temperature, pH and the presence of certain ions they fold into defined three-dimensional (3D) structures. When properly folded, these aptamers are able to bind other molecules (targets) with high affinity and specificity. Hydrogen bonding, hydrophobic and electrostatic interactions are the major chemical interactions, which lead to the high affinity of the aptamers to their respective targets [1,2]. Furthermore, the molecular recognition of aptamers is highly specific.

Aptamers are isolated via an *in vitro* selection process, which is called systematic evolution of ligands by exponential Enrichment (SELEX). A library containing 10^14^ to 10^15^ different randomized oligonucleotides is incubated with the target. Afterwards, unbound oligonucleotides are separated from the binding aptamer candidates. After amplification via PCR, the antisense strand is removed and the propagated aptamer candidates are subjected to the next round of selection and amplification. Usually, aptamer candidates have to compete for a limited amount of target. Therefore, aptamer candidates with low affinity are consecutively removed from the selection pool resulting in isolation of binders with high affinity after 6–12 SELEX rounds. Detailed reviews of the SELEX process, numerous improvements and variants of SELEX processes can be found elsewhere [3,4].

In comparison to antibodies, which are conventionally used in applications that require affinity ligands, aptamers feature comparable affinities and specificities. Beside this, aptamers are isolated and produced via *in vitro* operations and therefore exhibit low batch-to-batch variations. Furthermore, aptamers are long-term stable, even at elevated temperatures. These features make aptamers valuable affinity ligands which can be applied in several technologies including affinity chromatography [5], sensor platforms [6,7], and microarrays [8]. In this review we focus on aptamer microarray technology.

## 2. Aptamer Microarrays

### 2.1. Conventional Microarrays vs. Aptamer Microarrays

Classic DNA microarrays are used to measure mRNA levels thereby performing transcriptome analysis. In the initial step of a microarray experiment, the isolated mRNA is transcribed into cDNA, which is then fluorescently labeled and purified. The labeled antisense-orientated cDNA is then transferred to a microarray slide bearing immobilized complementary sense-orientated DNA oligonucleotide probes. Upon approaching the appropriate probe the cDNA hybridizes to this probe and is therefore immobilized. After the incubation, unbound cDNA is washed away and the relative frequency of remaining cDNA is estimated by measuring the fluorescence intensity of the features.

In complementary approaches protein microarrays allow the detection and quantification of proteins, which are the actual protagonists within living organisms. Protein microarrays are dominated by antibody microarrays, and are especially successful in the sandwich format. Immobilized antibodies capture their corresponding antigen, which is than detected by binding of a second fluorescently labeled detection antibody directed against a different epitope of the same antigen. Although these antibody‑based microarrays allow for ELISA-like detection of proteins in a multiplexed setup, there are still some limitations associated with this type of assay. Antibodies possess a rather low stability, resulting in limited shelf life and the need for cooled storage. Moreover, due to the development of antibodies in living organisms by immunization, not all antigens can be readily applied for antibody generation. This is especially true for small molecules, toxic substances and substances that are not able to provoke an immune response.

A suitable technology to overcome the limitations of protein/antibody microarrays is the aptamer microarray technology, which uses the advantages of DNA microarrays. Here, aptamers are immobilized on the microarray surface and serve as probes for various substances. As aptamers are also composed of nucleotides, well-known techniques for DNA microarray manufacturing can be used to produce aptamer microarrays. However, simply applying strategies of DNA microarrays to aptamer microarrays may not lead to adequate results. This discrepancy is a consequence of some special features of aptamers, which differ significantly from conventional DNA.

DNA microarrays are optimized for best possible DNA-DNA interaction. In contrast, aptamer microarrays not only consist of oligonucleotides, but also of analytes, which may be proteins or small molecules. These molecules can interact with other components than the aptamer (e.g., microarray surface). As a consequence, this may result in a high background signal which is lowering the performance of the aptamer microarray [9,10]. Surface properties can be adapted to this new requirement by applying suitable modifications. For example a PEGylation can be performed to reduce the unspecific binding of proteins to the surface [11,12]. The resulting coated surface is termed an antifouling matrix.

In contrast to “simple” base pairing of DNA strands, aptamers require a specific structure to bind their targets. Usually aptamers are able to fold into these defined 3D structures. However, the folding of an aptamer may be affected by aptamer immobilization, which can lead to a loss of functionality. Therefore it is much more difficult to optimize aptamer microarrays, and some general aspects have to be considered when developing an aptamer microarray. These aspects are elaborated in the following subsections.

### 2.2. Strategies for Aptamer Microarray Fabrication

Aptamer microarray fabrication techniques can be classified into two categories. On the one hand the aptamers can be synthesized directly on the microarray’s surface by *in situ* synthesis. This approach requires sophisticated synthesis machinery and is often used in serial production of commercial microarrays. However, there are some limitations, which arise when fabricating an aptamer microarray via *in situ* synthesis.

*In situ* synthesis yields in ready-to-use microarrays, therefore a post synthesis purification of aptamers is not possible. As a consequence, common synthesis procedures limit the aptamer length to 50 nt as for longer strands the given yield of full length aptamer is decreasing significantly [13]. In addition, aptamers may only be immobilized at their 3' terminus as oligonucleotides are synthesized from 3' to 5' and reverse synthesis direction is not established yet. Furthermore, single mutations within the aptamer sequence may lead to a full loss of function. Therefore, sequence fidelity is the most important parameter of *in situ* synthesis for aptamers [9]. More detailed information on *in situ* synthesis of aptamers (and oligonucleotides in general) can be found elsewhere [14].

On the other hand aptamers can be attached to the microarray after their synthesis and purification, so called post synthesis immobilization, on which we will focus in this section. Different strategies have been developed for the post synthesis approach. Usually, dissolved aptamers are spotted onto the microarray surface via contact or non-contact printing. Here, functionalized microarray substrates and modified aptamers are required to attach the aptamers to the surface.

### 2.3. Attachment Chemistries for Post Synthesis Immobilization

One possibility for aptamer immobilization is exploiting the streptavidin biotin interaction. In this case the microarray surface is coated with streptavidin and aptamers are labeled with biotin. When spotted on the array surface the biotin forms a stable complex with streptavidin and the aptamer is thereby immobilized. As the dissociation constant of the streptavidin-biotin complex is very low (10^−15^ M), and the complex is kinetically stable [15], this approach offers a fast, reliable and simple immobilization of biotinylated aptamers. Unfortunately streptavidin, like any other protein, can undergo denaturation. Therefore, aptamer microarrays based on streptavidin-coated slides degenerate over time even in chilled storage.

**Figure 1:**
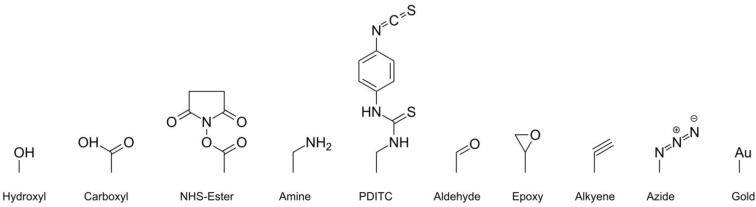
Different microarray surface modifications for aptamer immobilization.

To avoid this shortcoming and to produce microarrays taking advantage of the high stability of aptamers, aptamers can also be covalently immobilized directly on the microarray surface, which is usually a modified glass or polymer slide (see Figure 1 for the most common microarray modifications). Microarrays of this type can be stored at room temperature and are usually stable for several years. For the attachment of aptamers, different coupling chemistries have been developed.

The attachment chemistries described in the following display only a fraction of the diverse immobilization chemistries that are available. A more comprehensive review has been published by Balamurugan *et al.* [16].

Often simple chemical groups such as hydroxyl, carboxyl or amine are provided on the microarray’s surface. To allow immobilization of aptamers on these surfaces, aptamers can already be functionalized with different modifications during their synthesis. Conventionally amine or thiol modifications are utilized to immobilize oligonucleotides. The immobilization is often initialized by the addition of an auxiliary. Some slides are already activated by their manufacturers, so they do not need an auxiliary but are often sensitive to humidity.

The most basic type of surface function is the hydroxyl group. These slides allow the customers to apply their own surface chemistries. Often these slides are activated with carbonyldiimidazole (CDI) [17]. The formed carbamate is then attacked by a nucleophile, (amine or thiol group of modified aptamer) forming a covalent bond between the surface and the aptamer.

A common way to attach aptamers to a carboxyl-modified microarray surfaces is to activate the surface with 1-Ethyl-3-(3-dimethylaminopropyl)carbodiimide (EDC) and stabilize the intermediate with N-Hydroxysuccinimide (NHS). The resulting NHS ester is then reacting with an amine-modified aptamer to form a stable amide, which is connecting the aptamer to the microarray surface. The reaction can also be performed without using NHS. In that case, the formed intermediate is directly attacked by the amine-modified aptamer. This approach is not as efficient as using NHS, because the intermediate may also be hydrolyzed. When using NHS, the intermediate is transformed into a more stable NHS ester. The NHS ester is also subjected to hydrolysis but has a longer shelf life and therefore yields in higher immobilization efficiency [18,19,20]. Some manufacturers offer NHS activated slides in a hermetic sealing (e.g., Schott; Arrayit; PolyAn).

Amine-modified microarray substrates can be activated using p-Phenylene diisothiocyanate (PDITC). On the microarray, this symmetrical linker forms a thiourea structure. The remaining isothiocynanate group can react with another nucleophile such as an amine or thiol. Furthermore, thiolated aptamers can be attached to amine-modified surfaces using Sulfosuccinimidyl-4-(*N*-maleimidomethyl)cyclohexane-1-carboxylate (Sulfo-SMCC). On one site, this crosslinker bears an NHS ester, which is used to couple the molecule onto the amine-modified microarray surface. On the other site, the thiolated aptamer can react with the maleimide group in a thiol-Michael addition. Microarray slides with maleimide-activated esters are commercially available but should be used directly after the hermitic sealing is opened because the maleimide ester is susceptible to hydrolysis [21]. Another way to attach aptamers to amine-modified surfaces is using glutaraldehyde as a symmetrical linker. Together with the surface amine, glutaraldehyde forms an imine bond. The second aldehyde group reacts with an amine-modified aptamer in the same way. The imines should be reduced to amines with sodium borohydride or other reducing agents to improve the stability [22]. This principle can be transferred to attach amine-modified aptamers to an aldehyde-bearing surface.

Some manufacturers produce microarray slides with an epoxy-modified surface. Here, aptamers can be attached utilizing an amine or thiol modification, which reacts with the epoxy group via nucleophilic ring opening. This reaction does not require any auxiliary. Epoxy-modified microarrays are more stable to hydrolysis and have a longer shelf life than NHS ester-activated or PDITC activated microarray substrates.

A relatively new option for aptamer immobilization is the copper(I)-catalyzed azide-alkyne cycloaddition (CuAAC), a “click” reaction [23,24]. In this reaction an alkyne reacts with an azide forming a 1,2,3-triazole ring with respective ligands. For this approach the surface modification can either be azide or the alkyne, as aptamers can already be modified with either azides or alkynes. This approach features high immobilization efficiency and simple reaction conditions. However, up until now, only very few suppliers offer such surface modifications for microarrays.

Aptamers can also be immobilized on gold-coated surfaces. First, the aptamers are thiolated. Then, the thiol-group interacts with the gold surface via sulfur gold interaction resulting in immobilization of the aptamers on the surface. Nucleic acids can also stick to the surface by interaction of gold with the nitrogen side functions of the nucleobases [25]. Therefore, a second thiol-modified substance is often applied to substitute the adsorbed nitrogen from the gold surface, as the affinity of thiols to gold is higher than the affinity of nitrogen derivates [16].

### 2.4. Steric Requirements of Aptamers

Aptamers have certain steric requirements to attain their proper folding. These steric requirements can be challenged by different parameters during aptamer microarray production, which are discussed in the following.

#### 2.4.1. Surface Density

The surface density of the immobilized aptamers is an important parameter affecting aptamer microarray performance. If the surface density is low, this will result in a low signal from the feature and a limited dynamic range of the microarray. In contrast, if the surface density is too high, steric hindrance is suggested to cause problems in aptamer folding and therefore in subsequent binding of the target [26,27]. This will again result in a low signal.

One parameter that defines the microarray surface density is the loading capacity of the microarray surface. Different microarray substrates feature different loading capacities. In general, planar surfaces show a rather limited loading capacity. By applying a 3D layer onto the microarray surface, the available surface area is greatly enhanced, which yields in a higher loading capacity. However, 3D microarray substrates tend to exhibit higher background fluorescence [28].

The surface density is not only dependent on the loading capacity, but also on the spotting concentration of the aptamer and the immobilization efficiency. Consequently, the aptamer density can be easily controlled via variation of the aptamer concentration. Moreover, print buffers and additives within the print buffers may affect the immobilization efficiency [29].

#### 2.4.2. Surface Charge

Oligonucleotides are negatively charged and tend to stick to positively charged surfaces. This electrostatic interaction interferes with the proper folding of the immobilized aptamer and may lead to a loss of functionality. The surface charge can be reversed by capping the surface with a negatively charged agent [30] resulting in a repulsion of the aptamers by the capped surface. On the other hand, many aptamers require divalent ions (e.g., magnesia, calcium) for their correct folding. These ions can mediate electrostatic interactions between a negatively charged surface and the aptamers nucleotides [31].

#### 2.4.3. Proximity to Surface and Spacers

Steric requirements of aptamers may be challenged if the aptamers are immobilized too close to the microarray’s surface [30]. This effect varies for each aptamer and has to be investigated empirically. While some aptamers stay functional when directly attached to the surface, others undergo significant conformal changes and lose their functionality. Furthermore, the orientation in which the aptamer is immobilized affects the folding of the aptamer. Again there are aptamers that stay functional when immobilized via either 5' or 3' terminus and there are aptamers, which lose their function when immobilized via the “wrong” terminus. There are also cases in which an aptamer loses its function independent from the orientation in which it is immobilized. No general rule can be given for the best strategy to immobilize aptamers in a functional state, but to investigate these parameters for each aptamer individually.

To overcome problems in aptamer folding caused by surface proximity, molecular spacers are often inserted between the aptamer and the microarray surface. These spacers bear a reactive group, which is used to couple the aptamer onto the surface. When attached to the microarray, the spacer spans a certain distance allowing the aptamer to fold correctly.

Usually linear spacers such as poly(dT) or carbon chains are attached to the aptamer before immobilization. Also some polymers like polyethylene glycol or polyethylene imine have been used to immobilize aptamers. Table 1 gives an overview over the properties of the most common spacer types for aptamer immobilization.

**Table 1:** Different spacer types applied for aptamer immobilization and their basic properties.

Spacer type	Abbreviation	Length/unit [angstrom]	Hydrophobicity
Aliphatic carbon chain	C	1.57 [32]	Hydrophobic
Polyethylene glycol	PEG	3.51 [33]	Hydrophilic
Polyethylene imine	PEI	2.9–3.5 [34]	Hydrophilic
Poly thymine	poly(dT)	3.4 [35]	Hydrophilic

To simultaneously enhance the loading capacity of the surface and the distance between aptamer and the microarray surface, nonlinear, branched spacers or dendrimers can be applied to immobilize aptamers. Once attached to the surface, these molecules show multiple sites for aptamer immobilization.

As simple as the idea of using a spacer may be, there are several effects that may occur when using a spacer within aptamer microarrays. Even though long spacers are used to immobilize aptamers, they may not remain fully functional when immobilized. The correct folding of the aptamer can be suppressed by the spacer itself due to steric hindrance or other interactions with the aptamer. If a long spacer is applied, this spacer may be irregularly folded itself, which again could challenge the steric requirements of the corresponding aptamer [36].

Again, the spacer position within the aptamer has a significant effect on the folding and function of an aptamer. The strength of this effect differs from aptamer to aptamer [10,27,30,36]. However, the preferred orientation of an aptamer may be predicted by atomic scale calculation [37]. Similar effects on the folding of an aptamer can also be observed when introducing other modifications like fluorescent dyes to an aptamer [8].

A spacer may also affect the efficiency of aptamer immobilization. Edwards *et al.* claimed that elongated poly(dT) spacers lower the immobilization efficiency, possibly due to a charge barrier [27]. On the other hand this reduced immobilization efficiency may leave more space for the aptamers to fold into their active structure and therefore result in a higher ratio of functional aptamers in the feature [35].

### 2.5. Assay Formats

**Figure 2:**
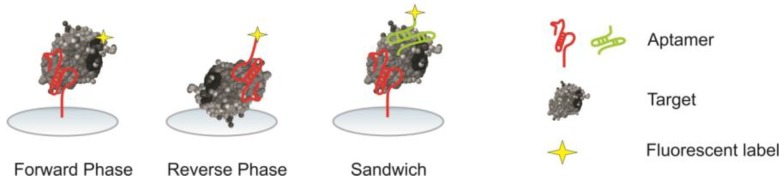
Schematic overview over different aptamer microarray formats.

Aptamer microarrays can be performed in different formats (phases), see Figure 2. A literature overview is given in Table 2.

#### 2.5.1. Forward Phase

In a forward phase microarray, the recognition element, namely the aptamer, is immobilized on the surface. At the beginning of incubation the analytes are free in solution; later, they are captured by the aptamer. Except for label-free detection techniques like SPR, in this assay format the analyte has to be labeled, which may interfere with subsequent analyte capture by the aptamer [10]. Moreover, if components in the sample form complexes with the target, these complexes may be detected at the specific feature. As the non target compounds also increase the given signal from the feature, this leads to an overestimation of the target concentration and therefore to false results in a forward phase microarray [38].

By immobilizing more than one aptamer on the aptamer microarray, a forward phase microarray can give insight into many conditions within a single sample. When analyzing more samples, additional microarrays have to be performed. However, by partitioning the microarray into subarrays several samples can be investigated in parallel (e.g., by using the 16 pad format).

#### 2.5.2. Reverse Phase

In contrast to the forward phase microarray, in a reverse phase microarray, the analyte is immobilized on the microarray surface. This immobilization can either be based on adsorptive interactions or covalent attachment. An aptamer is used to detect the analyte within the sample. A highly parallel detection of the same analyte in different samples is possible when immobilizing these samples on the same slide.

This approach has mainly been applied to proteins, which then is called a reverse phase protein microarray or lysate microarray. In contrast to antibodies, which must be labeled after purification, aptamers can be labeled during their synthesis in a site-specific manner. This circumvents possible uncontrolled losses of function during labeling. In comparison to antibodies, which are usually applied as the recognition element, aptamers feature a higher signal to noise ratio and an improved limit of detection [8]. Besides these advantages of aptamer-based reverse phase microarrays, their applicability is rather limited. When spotting a complex sample containing only a small portion of the target to be analyzed, this target has to compete with other proteins for free immobilization sites on the microarray´s surface. Consequently, only small amounts of the target are immobilized resulting in low signals and low sensitivity of the microarray, especially when complex samples are applied.

#### 2.5.3. Sandwich

A sandwich microarray is a modified forward phase microarray in which the analyte is not labeled by itself, but addressed by another aptamer carrying the label for detection. However, to perform an assay of this type, the bound target has to display a second epitope, and even more importantly, two aptamers binding to different epitopes of the target have to be available.

In comparison to a reverse phase microarray, this approach is more sensitive [10]. During the incubation of the sample the analyte is captured by the aptamer and therefore concentrated within the spot. The loading of the analyte within the spot is much higher in a sandwich assay, because in a reverse phase microarray mostly complex samples are immobilized, while in the sandwich format the target is affinity purified by the immobilized aptamer. Furthermore, a sandwich assay exhibits higher specificity than an assay with a single affinity ligand (including forward phase and reverse phase microarray), as the target must be recognized by two affinity ligands rather than by one to result in a signal. This also reduces problems that rise from analyte-complexes.

### 2.6. Detection Methods

To measure the aptamer target interaction different detection methods can be applied to aptamer microarrays. The most common methods are described in the following.

#### 2.6.1. Fluorescent Dye-Based

Like in classic DNA microarrays, fluorescent dyes can be applied in aptamer microarrays, as aptamers can be labeled site specifically with dyes during their synthesis; this simplifies setting up a reverse phase or sandwich aptamer microarray. When performing a forward phase microarray the analyte has to be labeled. Depending on the substance, modern coupling chemistry provides a versatile toolbox for attaching a fluorescent dye to a given target. The use of a fluorescent dye therefore is compatible with all assay formats. The easy access to fluorescent conjugates and the fact that the required instrumentation for detection is the same as in conventional microarrays make this the most frequently used detection method (see Table 2).

In a forward phase microarray, the targets (mostly proteins) are often labeled before incubation with the aptamer microarray. The excessive unreacted dye is then removed and the target/dye ratio is determined as this ratio is an important parameter, which significantly affects the microarray performance. If the ratio is too low, this will result in low feature signal. On the other hand, extensive labeling may result in targets whose epitopes are masked by the label and therefore may not be recognized by the aptamer anymore [10,39]. To circumvent this limitation, staining can be done after capture with a fluorescent reagent specific for a class of targets (e.g., universal protein stain [40,41]).

It should be noted that the fluorescence scanner and the scanning settings have a significant impact on the resulting statistic variations and microarray performance [42].

#### 2.6.2. SPRi

Surface plasmon resonance (SPR) has been extensively used to determine kinetics and binding affinities. Due to further development of this technology, it also possible to measure the resonance at each place within a whole chip in real time (surface plasmon resonance imaging (SPRi)). As a consequence, these chips can serve as substrates for multiplex analysis and can be applied to aptamer microarrays as well. This method is suitable for any assay format, as the measuring principle does not require a label, neither on the target nor on the aptamer. However, for a measurement either the aptamer or the target has to be immobilized on the chip surface. Due to matrix effects and leakage of molecules immobilized by adsorption, the baseline may shift during the measurements [43]. Furthermore, detection of small molecules is challenging as they generate only a small signal [44].

#### 2.6.3. Other

Beside the methods described above, microarrays can also be read by using a radiolabel, via electro-chemical detection [6,45] or by *matrix-assisted laser desorption/ionization* (*MALDI*) mass spectrometry [46,47,48]. Furthermore, alternative label-free detection methods are emerging [49]. These methods have gained much attention as they do not require a label which would interfere in the folding of the aptamer or the interaction with the target. However, label-free detection relies on the intrinsic properties of an analyte. As there are many different techniques, label-free detection methods are reviewed elsewhere [50].

### 2.7. Applications of Aptamer Microarrays

To date, aptamer microarrays are almost exclusively applied to proteins (see Table 2). The quantification of small molecules or toxins is still dominated by classic methods like HPLC or GC. As a substitute, aptasensors offer a rapid detection of a single analyte within the sample. However, multiplexed measurements of various proteins are of greater interest. Consistently, aptamer microarrays are currently focused on the quantification of proteins. Efficient immobilization and labeling technologies facilitate easy access to the microarray format.

A novel application of aptamer microarrays is the investigation of protein-protein interactions (PPI) [38]. Here, immobilized aptamers are used to capture their target. The target is labeled and the given signal is used as a control. Then, a complex protein sample (e.g., cell lysate) with a different label is incubated with the microarray. In case of a protein-protein interaction the second label can be detected on the feature after the washing steps.

Another promising field of application for aptamer microarrays is medicine. SomaLogic has developed different enhanced aptamer microarrays for the discovery of potential biomarkers and has demonstrated their successful use [51,52]. In the future aptamer microarrays may also be used to analyze patient samples on the presence or elevated levels of biomarkers [47,53].

Furthermore, microarrays offer new possibilities for the evaluation and selection of aptamers. Microarrays have been used to analyze aptamer candidates which have been isolated in a SELEX procedure [39]. Several immobilized aptamer candidates are incubated with labeled target, and the affinity of the candidates is estimated by the resulting signal. In addition, this approach can be used to determine the binding site of an aptamer by immobilizing and assaying different truncated variations of this aptamer [54,55,56]. It is also possible to optimize a given aptamer by generation and microarray‑assisted evaluation of mutated sequences [57]. Furthermore, the complete selection of an aptamer can be performed on chip as has been demonstrated by Knight *et al.* [58] and Platt *et al.* [59]. First, a library of randomized sequences was generated *in silico*, which then was synthesized on a microarray at distinct places. After the microarray was incubated with a given target, the binding on each feature was measured and new sequences for the next selection round were generated via a genetic algorithm. In this way aptamers with high affinities were generated (approx. 1.7–5.0 nM [58]; 24 nM respectively [59]).

**Table 2:** Overview of aptamer microarray publications.

Target Name	Assay-Format	Multiplexing	Detection technique	Limit of detection	Author + Year
Toll-like receptor 2	forward		fluorescence scanner		Chang 2009 [54]
Thrombin/ E. coli total protein	forward	12	fluorescence scanner		Chen 2013 [38]
Human Thrombin/VEGF	forward	2	SPR		Chen 2012 [60]
4 different proteins	forward	2 × 2	fluorescence scanner	pM–nM	Cho 2006 [10]
Human angiopoietin-2	forward	15.000	fluorescence scanner		Cho 2013 [61]
Lysozyme; IgE	forward	4	fluorescence scanner	70 fM; 5.2 fM	Collett 2005 [62]
Lysozyme	forward	26	fluorescence scanner	70 fM	Collett 2005 [39]
Thrombin	forward		SPR	100 pM	Daniel 2013 [63]
IgE	forward	15.000	fluorescence scanner		Fischer 2008 [55]
Streptavidin	forward	yes (?)	fluorescence scanner		Franssen-van Hal 2013 [9]
Thrombin	forward	2	fluorescence scanner	30–50 pM	Lao 2009 [35]
human fIXa	forward	5	SPR	10 nM	Li 2006 [64]
4 different proteins	forward	4	fluorescence polarization		McCauley2003 [65]
Thrombin	forward	4.6 × 10^4^	fluorescence scanner		Platt 2009 [57]
PFEI-His	forward		fluorescence scanner		Sinitsyna 2012 [28]
BGL-His + streptavidin	forward		fluorescence scanner		Walter 2008 [30]
IgE; IgG	forward		SPRi	2 nM	Wang 2007 [66]
PFEI-His	forward		fluorescence scanner		Zhu 2011 [36]
17 different proteins	forward/heterogenic sandwich	17	fluorescence scanner	pM–nM	Bock 2004 [41]
Thrombin	forward/sandwich		resonance waveguide diffraction; fluorescence reader		Edwards 2010 [27]
IgE	heterogenic sandwich		SPR	1 fM	Kim 2010 [67]
Thrombin / VEGF	heterogenic sandwich	4 + 1 Control	SPR	1 pM (VEGF)	Li 2007 [68]
PFEI-His	reverse		fluorescence scanner	30 nM	Lübbecke 2012 [8]
HCVNS3 protein	reverse		confocal laser scanning microscope	73 pM	Roh 2010 [69]
Yeast TBP (TATA Binding Protein)	sandwich		fluorescence scanner	nM–µM	Ahn 2010 [47]
Thrombin	sandwich		fluorescence scanner	64 pM	Edwards 2010 [70]
Thrombin	sandwich		fluorescence scanner	0.17/0.75 nM	Meneghello 2012 [71]
8 different proteins	sandwich	8	dual laser flow system; fluorescence	1–100 pM	Ochsner 2014 [72]
C-reactive protein (CRP)	sandwich		fluorescence scanner	43 pM	Pultar 2009 [29]
Thrombin + VEGF	sandwich	2	fluorescence scanner	50 nM	Sosic 2013 [73]
Thrombin	sandwich		fluorescence scanner		Sosic 2011 [74]
Thrombin	sandwich		fluorescence microscope	0.27 nM	Tennico 2010 [75]
Ethanolamine	pseudo-sandwich (TID)		fluorescence scanner	10 pM	Heilkenbrinker 2014 [76]

### 2.8. Current Limitations and Future Prospects

Many experiments have been performed to show that aptamers can be applied successfully within different microarrays formats (Table 2). Furthermore, there are very few commercial versions of aptamer microarrays available already (SOMAscan™). New detection techniques are emerging and current technologies are further developed to allow multiplexed analysis. However, there is still a lack of aptamers that are suitable for microarrays. While aptamers with rather high K_D_ (in µM rage) have been successfully used in different applications like affinity separation [5,77], aptamer microarrays require aptamers with low binding constants [41]. Also the aptamer target complex should be slow dissociating [78]. This is especially important for microarrays, as an extension of washing steps can increase the signal to noise ratio but excessive washing should not result in a loss of aptamer-target complex.

Other issues are associated with the applied assay conditions, as many of the aptamers have been selected by different scientists and under different conditions. Therefore, different aptamers require different buffers, additives, ions and ion strengths for their correct folding and function [10,70]. Trying to assemble a multiplex microarray by matching these requirements is quite challenging. When selecting aptamers that should be active together, it is best to select them under the same conditions.

In a sandwich microarray, aptamers are required which bind to different epitopes of the target as the target is addressed by two aptamers. Aptamer pairs that exhibit this behavior may be found by incident. As a more constructive approach, adapted SELEX procedures have been developed to select aptamer pairs which address different epitopes [79].

Especially in the context of protein detection, aptamer microarrays have to compete with well-characterized antibody microarrays. In comparison to antibodies, aptamer technology is relatively new. The industry has put lots of efforts and money into the development of antibodies for the most important targets. Selecting aptamers against these targets would be a great outlay resulting in additional resistance to novel aptamer-based techniques.

Microarrays for the detection of proteins are therefore largely dominated by antibodies. However, the use of antibodies for the detection of small molecules is limited as the generation of an antibody for a small molecule is difficult, since the target has to be immunogenic and must only exhibit a moderate toxicity. Furthermore, antibodies are about six times bigger than aptamers and cannot be used in sandwich assays for small molecules, which do not provide enough surface area for the simultaneous binding of two antibodies. On the other hand, it is possible to isolate aptamers against nearly any given target. Therefore aptamers can also be generated against small molecules, which are not immunogenic. Although some general problems arise when selecting an aptamer against a small targets, including the change of the target structure during immobilization and difficulties in determination of binding constants, aptamers have already been selected against a broad range of different small molecules [80]. New SELEX techniques like Capture SELEX allow for the screening of aptamers against small molecules without the need to immobilize the target. This has been recently demonstrated by Strehlitz and coworkers by the selection of aptamers targeting antibiotics [81]. These new techniques can be expected to further accelerate the use of aptamers in the context of small molecule detection.

When applying classic microarray formats to small molecules using aptamers, some critical aspects have to be considered. If performing a forward phase assay the target has to be labeled. This may cause problems because the overall molecular structure is significantly changed by labeling a small molecule with another molecule with equal or greater size. In a reverse phase microarray, the small target molecules have to be immobilized, which may also cause problems in subsequent aptamer binding.

As a sandwich microarray would grant a label-free detection, it would therefore be a good alternative for small molecules. But aptamers often enclose their small molecule target by folding “around” it. The target would not display a second epitope for the second aptamer. In this case—as already described for antibodies—a sandwich assay is not possible.

In this context, aptamers offer new assay designs, which could not be realized with antibodies, to overcome these limitations. Target-induced dissociation of complementary oligonucleotides (TID) can be used to detect small molecules with an aptamer microarray [6,76]. A fluorescent-labeled antisense oligonucleotide is hybridized to an immobilized aptamer prior to the incubation with the target. The fluorescence of the feature is measured. In presence of the target, the aptamer oligonucleotide hybrid dissociates, the antisense oligonucleotide is replaced by the target and the fluorescence of the feature is therefore lowered. We have recently demonstrated the applicability of TID for microarray-based detection of small molecules using an aptamer directed against ethanolamine. This strategy resulted in an excellent sensitivity (LOD = 10 pM) [76].

Another detection mechanism that could be useful in the aptamer-based detection of small molecules is target-induced structure switching (TISS) of aptamers [6]. During the binding of a target, an aptamer undergoes conformal changes. These changes may be measured by electrochemical detection or in an aptamer beacon format. In the latter, a fluorophore and a quencher are attached to the aptamer. Depending on the mode of the beacon, conformal changes may either approximate or depart the fluorophore and the quencher. In this way a measurable signal is generated with only one aptamer. While until now TISS-based detection was only performed in solution, it could also be transferred to microarray surfaces.

A different option for small molecule detection may be the target-induced reassembly (TIR) of aptamers [6]. Here an aptamer fragment could be immobilized on the microarray surface. The microarray is then incubated with a mixture of the sample and a solution containing the second fragment of the aptamer. In presence of the target a complex of the two aptamer fragments and the target is formed. This pseudo-sandwich assay seems to be especially advantageous for the detection of small molecules. Although this principle has only been applied on a bead matrix, it is a promising approach for the detection of small molecules, which should be further investigated.

## 3. Conclusion and Outlook

It has been demonstrated that aptamers have been successfully applied as affinity ligands in many different techniques. Here, aptamers exhibit a high affinity and specificity. Aptamers can be selected against virtually any given target. Furthermore, automated and high throughput selection strategies and platforms have been developed to overcome the lack of suitable aptamers for a variety of different applications. Due to their chemical synthesis aptamers are relatively cost-efficient and show minimal batch-to-batch variations. Therefore, well-selected aptamers are valuable and reliable affinity ligands. Aptamers also allow highly multiplexed analysis, if the aptamers are selected under the same conditions. As a consequence, aptamers show great potential in microarray development.

To date aptamer microarrays are focused on the detection of proteins, but alternative assay formats also allow the detection of small molecules. Therefore, aptamer microarrays could be especially useful in the detection and quantification of small molecules. We expect them to become powerful tools for the easy and multiplexed profiling of small molecules, thereby opening up new applications, including metabolomics.
